# Synaptojanin 1 Modulates Functional Recovery After Incomplete Spinal Cord Injury in Male Apolipoprotein E Epsilon 4 Mice

**DOI:** 10.1089/neur.2023.0023

**Published:** 2023-07-27

**Authors:** Carlos A. Toro, Jens Hansen, Mustafa M. Siddiq, Kaitlin Johnson, Jiqing Cao, Adriana Pero, Ravi Iyengar, Dongming Cai, Christopher P. Cardozo

**Affiliations:** ^1^ Spinal Cord Damage Research Center, James J. Peters VA Medical Center, Bronx, New York, USA.; ^2^ Neurology Service, James J. Peters VA Medical Center, Bronx, New York, USA.; ^3^ Research and Development, James J. Peters VA Medical Center, Bronx, New York, USA.; ^4^ Department of Medicine, Icahn School of Medicine at Mount Sinai, New York, New York, USA.; ^5^ Department of Pharmacological Sciences, Icahn School of Medicine at Mount Sinai, New York, New York, USA.; ^6^ Department of Rehabilitative Medicine, Icahn School of Medicine at Mount Sinai, New York, New York, USA.; ^7^ Department of Neurology, Icahn School of Medicine at Mount Sinai, New York, New York, USA.

**Keywords:** contusion SCI, functional recovery, PIP_2_, Synj1

## Abstract

Apolipoprotein E epsilon 4 (ApoE4) is the second most common variant of ApoE, being present in ∼14% of the population. Clinical reports identify ApoE4 as a genetic risk factor for poor outcomes after traumatic spinal cord injury (SCI) and spinal cord diseases such as cervical myelopathy. To date, there is no intervention to promote recovery of function after SCI/spinal cord diseases that is specifically targeted at ApoE4-associated impairment. Studies in the human and mouse brain link ApoE4 to elevated levels of synaptojanin 1 (synj1), a lipid phosphatase that degrades phosphoinositol 4,5-bisphosphate (PIP_2_) into inositol 4-monophosphate. Synj1 regulates rearrangements of the cytoskeleton as well as endocytosis and trafficking of synaptic vesicles. We report here that, as compared to ApoE3 mice, levels of synj1 messenger RNA and protein were elevated in spinal cords of healthy ApoE4 mice associated with lower PIP_2_ levels. Using a moderate-severity model of contusion SCI in mice, we found that genetic reduction of synj1 improved locomotor function recovery at 14 days after SCI in ApoE4 mice without altering spared white matter. Genetic reduction of synj1 did not alter locomotor recovery of ApoE3 mice after SCI. Bulk RNA sequencing revealed that at 14 days after SCI in ApoE4 mice, genetic reduction of synj1 upregulated genes involved in glutaminergic synaptic transmission just above and below the lesion. Overall, our findings provide evidence for a link between synj1 to poor outcomes after SCI in ApoE4 mice, up to 14 days post-injury, through mechanisms that may involve the function of excitatory glutaminergic neurons.

## Introduction

Spinal cord injury (SCI) results in devastating and lifelong disabilities. Approximately two thirds of persons with SCI eventually recover some sensory and motor function.^1^ The extent of this recovery is predicted by and correlated with the amount of spared white matter at the lesion site.^2^ Mechanisms for recovery are thought to involve remyelination of surviving axons, neuroplasticity within the spinal cord and brain,^3–5^ and activities of microglia and astrocytes, which may promote or reduce locomotor function.^6–9^ Considerable evidence indicates that the neuroanatomical basis for motor functional recovery is largely dependent upon axon branches arising above the site of axon transection by the SCI. These branches synapse with neurons located in the brainstem and in gray matter of the spinal cord that are above the lesion site and project descending axons past the lesion.^3–5,10^

Although genetic variability is recognized as a key determinant of risks for common diseases from cancer to cardiovascular disease and sporadic Alzheimer's disease (AD), there is very little knowledge available as to how naturally occurring genetic variability influences outcomes after SCI. One genetic variant that may determine outcomes after SCI is apolipoprotein E (ApoE) epsilon 4 (ApoE4). ApoE is synthesized by hepatocytes, astrocytes, and microglia and carries lipids from these cells to target cells elsewhere in the body. In humans, three ApoE variants have been well characterized and are distinguished by their amino acid sequence. ApoE2 has cysteine residues at positions 112 and 158; ApoE3 has one substitution (Cys158Arg), and ApoE4 has two (Cys112Arg, Cys158Arg).^11^ The worldwide prevalence of these variants is 8.4% (ApoE2), 77.9% (ApoE3), and 13.7% (ApoE4), respectively.^11^ The ApoE4 variant has been associated with health problems that include sporadic AD,^12^ worse outcomes after traumatic brain injury,^13,14^ and greater risks of kidney and cardiovascular disease.^15,16^ By contrast, ApoE2 seems to protect against cognitive decline.^17–19^

A growing body of evidence links ApoE4 to poor outcomes after SCI or spinal cord disease. Analysis of a case series of newly injured SCI patients found that those who carried at least one ApoE4 allele had longer inpatient rehabilitation stays and recovered less function based on standard neurological exams.^20^ The idea that ApoE4 is associated with less functional recovery after SCI gained further support from prospective studies of patients with cervical myelopathy, a neurological syndrome caused by spinal cord compression, which showed that patients who carried ApoE4 were much less likely to recover function after surgical decompression of the cervical spinal cord.^21^ Parallel studies using a mouse model of cervical myelopathy found that, as compared to ApoE3, ApoE4 impaired the recovery of forelimb grip strength after cervical decompression associated with increased activation of microglia, reactive astrocytes, and expression of messenger RNA (mRNA) for proinflammatory cytokines.^21^

We recently found that, when compared to ApoE3 mice, ApoE4 mice demonstrated lower locomotor function after a moderate-severity contusion SCI; ApoE4 was associated with higher expression in spinal cord of markers for reactive astrocytes (glial fibrillary acidic protein) and activated microglia (ionized calcium-binding adapter molecule 1),^22^ but, importantly, did not influence white matter sparing after SCI,^22^ indicating that ApoE4 most likely negatively impacts the function of surviving neural circuits or formation of relay circuits connecting the brain and periphery.

Synj1 is one of a growing number of genes implicated in the adverse effects of ApoE4 on cognitive function with aging.^23^ Synj1 is critical for endocytosis and trafficking of synaptic vesicles and autophagosomes,^24^ and for rearrangements of the cytoskeleton. These actions are thought to be largely attributable to the dephosphorylation by synj1 of phosphoinositol 4,5-bisphosphate (PIP_2_) to inositol 4-monophosphate (PIP_1_).^25^ Synj1 expression is increased in the brains of humans and mouse ApoE4 carriers of mild cognitive impairment and early AD associated with reduced levels of PIP_2_ in tissue homogenates and synaptosomes, and the genetic reduction of synj1 rescued AD-related cognitive impairments in ApoE4 mice.^23^ Whether Synj1 could modulate recovery after SCI is not known.

The goal of the experiments reported here was to investigate the role(s) of synj1 in locomotor function recovery after SCI in ApoE4 mice up to 14 days post-injury. We tested whether, as compared to ApoE3, ApoE4 was associated with higher synj1 expression and lower PIP_2_ levels in spinal cord tissue, and whether genetically reducing synj1 would improve locomotor function after a contusion SCI in ApoE3 or ApoE4 mice. Potential cellular and molecular effects of the genetic reduction of synj1 after SCI were further investigated by reverse-transcription (RT)/quantitative (qPCR) polymerase chain reaction (PCR) and bulk RNA sequencing (RNA-seq).

## Methods

### Animals

Use of live animals was conducted in accordance with Public Health Service Policy on Humane Care and Use of Laboratory Animals and the Guide and was approved by the Institutional Animal Care and Use Committee at James J. Peters Veterans Affairs Medical Center. Mouse models expressing human ApoE3 and ApoE4 variants with either synj1^+/+^ or synj1^+/–^ backgrounds were bred as previously described.^23^ Genotypes were verified by PCR amplification.

### Experimental design

**FIG. 1.**
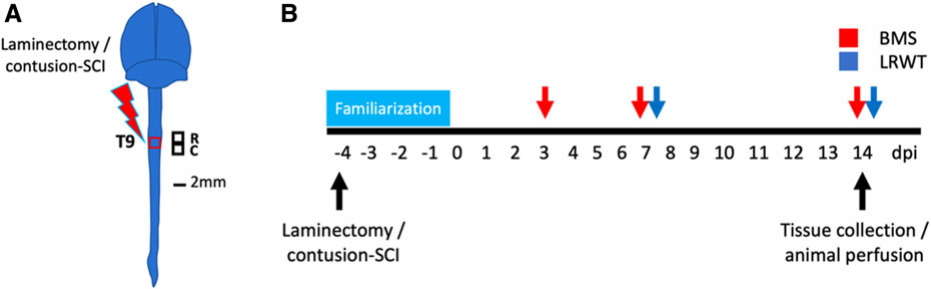
Experimental design. (**A**) Anatomical representation of the mouse brain and spinal cord showing the site of laminectomy or contusion SCI (red box) at the level of thoracic vertebrae 9 (T9). Black squares represent locations of the rostral and caudal segments of spinal cord collected for histology, biochemistry, and RNA-seq studies. R = rostral; C = caudal. Scale bar, 2 mm. (**B**) Timeline for animal familiarization with handling, behavioral testing, and tissue collection/animal perfusion are depicted. BMS, Basso mouse scale; LRWT, ladder rung walk test; RNA-seq, RNA sequencing; SCI, spinal cord injury .

At 4 months of age, male mice were randomly assigned to experimental groups (Table 1). The experimental design and timeline for the study procedures are shown in Figure 1.

**Table 1.** Experimental Groups and Animal Numbers

***Surgery***	***Behavior***	***Histology***	***HPLC***	***Biochemistry***	***RNA-seq***	***Tissue collected***
Laminectomy (sham)/ApoE3 Synj1^+/+^	*N* = 6	—	*N* = 6	—	—	*N* = 6
Laminectomy (sham)/ApoE3 Synj1^+/–^	*N* = 6	*N* = 3	—	*N* = 3	—	*N* = 6
Laminectomy (sham)/ApoE4 Synj1^+/+^	*N* = 6	—	*N* = 6	—	—	*N* = 6
Laminectomy (sham)/ApoE4 Synj1^+/–^	*N* = 6	*N* = 3	—	*N* = 3	—	*N* = 6
Contusion SCI/ApoE3 Synj1^+/+^	*N* = 9	*N* = 4	*N* = 6	*N* = 6	*N* = 3	*N* = 16
Contusion SCI/ApoE3 Synj1^+/–^	*N* = 6	*N* = 2	—	*N* = 5	*N* = 3	*N* = 7
Contusion SCI/ApoE4 Synj1^+/+^	*N* = 10	*N* = 4	*N* = 6	*N* = 4	*N* = 3	*N* = 14
Contusion SCI/ApoE4 Synj1^+/–^	*N* = 11	*N* = 4	—	*N* = 7	*N* = 3	*N* = 11

ApoE3, apolipoprotein E epsilon 3; synj 1, synaptojanin 1; ApoE4, apolipoprotein E epsilon 4; SCI, spinal cord injury; HPLC, high-pressure liquid chromatography; RNA-seq, RNA sequencing.

### Spinal cord injuries

A moderate-severity contusion SCI was performed using the Infinite Horizons (IH) impactor (Precision Systems and Instrumentation, LLC, Fairfax Station, VA), as reported previously.^22^ Induction of anesthesia was performed by 3% isoflurane inhalation. Animals at 4 months of age and similar weights (Supplementary Fig. S1) underwent a laminectomy to expose the dura at the level of T9. The laminectomy of ∼2 mm in diameter allowed the impact probe to hit the dura without touching any bone or other surrounding tissues. Stabilization of the vertebral column was done using forceps attached to the IH clamping platform. All animals in the SCI groups (Table 1) received a 50-kdyne contusion.^26^ Left-right lesion symmetry was confirmed by detection of bruising on both sides of the dorsal median sulcus. Actual impact force and cord displacement values were recorded for each animal (Supplementary Fig. S2). Muscle was closed using resorbable sutures and skin closed with 7-mm wound clips. Post-operative care took place over heating pads warmed with recirculating water. All animals received pre-warmed lactated Ringer's solution, carprofen, and Baytril for the first 5 days after surgery. Urine was expressed manually twice a day by gentle pressure and massage of the bladder until spontaneous voiding was observed. Animals were checked for retained urine and overall health at least once a day for the length of the study.

### Behavioral testing

Locomotor recovery was tested as previously reported,^22^ using the Basso Mouse Scale (BMS) open-field test^27^ and the horizontal ladder rung walk test (LRWT),^28^ pre-operatively and at specified time points after SCI (Fig. 1). BMS scores are a well-established method for evaluating the severity of impairments in locomotor function after SCI. LRWT evaluates fine motor skills and coordinated stepping.^28^ Animals were recorded as they attempted to cross the ladder. Videos were reviewed by two blinded observers who recorded the number of correct steps and foot-placement errors. All animals were familiarized with the equipment 1 week before surgery.

### Tissue harvest

At 14 days post-SCI, 4 animals per group (Table 1) were randomly selected to undergo perfusion fixation under a deep anesthesia achieved by intraperitoneal injection of ketamine (100 mg/kg) and xylazine (30 mg/kg), as previously reported.^22^ Mice were euthanized by transcardial perfusion with sterile saline followed by ice-cold 4% paraformaldehyde (PFA). Perfused spinal cords were removed and post-fixed in 4% PFA for 72 h, transferred to a solution of 30% sucrose, and stored at 4°C until cryostat sectioning. Additional fresh-frozen spinal cord tissues from each group were collected after inducing deep anesthesia by inhalation of 3% isoflurane followed by decapitation. Spinal cord segments, immediately rostral or caudal from the lesion epicenter, were collected and snap-frozen in liquid nitrogen and then stored at −80°C for biochemical analysis; tissues from comparable areas of spinal cords of sham-controls were also collected.

### Immunofluorescence staining

Transverse 10-micron cryosections of perfusion-fixed spinal cord were used for immunofluorescence staining as previously reported.^22^ Antibodies were used to detect PIP_2_ and synj1. Secondary antibodies were Alexa Fluor 488 and Alexa Fluor 647 (Table 2). Sections were imaged with a confocal microscope (Zeiss LSM700; Carl Zeiss, Jena, Germany) and analyzed using ImageJ software (National Institutes of Health, Bethesda, MD), as detailed below.

**Table 2.** Antibodies for Immunohistochemistry

***Antibody name***	***Isotype and host***	***Catalog no.***	***Concentration***	***RRID***
Anti-Synj1 antibody	IgG polyclonal, host rabbit	Novus, NBP1 87842	1:1000	AB_443303
Anti-PIP_2_ antibody	IgG polyclonal, host rabbit	Echelon, Z-P034	1:400	AB_305808
Alexa-488 anti-mouse	IgG polyclonal, host goat	Abcam, ab150113	1:2500	AB_2636859
Alexa-647 anti-rabbit	IgG polyclonal, host goat	Abcam, ab150079	1:2500	AB_2576208

Synj 1, synaptojanin 1; PIP_2_, phosphoinositol 4,5-bisphosphate; IgG, immunoglobulin G.

### White spared matter detection

Transverse 10-micron cryosections of perfusion-fixed spinal cords were obtained using a cryostat (Leica Microsystems, Wetzlar, Germany), and immunofluorescence staining was performed as previously reported.^22^ Myelin was stained using Fluoromyelin, according to the manufacturer's protocol (FluoroMyelin green; ThermoFisherScientific, Waltham, MA). We then analyzed spared myelin in sections obtained at 150 microns both rostral and caudal of the injury site and at the epicenter. Sections were imaged with a confocal microscope (Zeiss LSM700; Carl Zeiss) and analyzed using ImageJ software (National Institutes of Health). Visualization of differences in spared myelin and image analysis was performed as previously described.^29^ Briefly, thresholds for signals in the images were set at background gray values. The region of interest, defined by a dotted line, was determined and the mean intensity and total number of pixels above threshold were measured. Spared tissue was determining by the percentage of white matter calculated by the total area of the spinal cord for each section using ImageJ.

### Image capture and quantification

Tiled images (5 × 5) were obtained from stained sections using a 20 × objective and a Zeiss LSM700 confocal microscope (Carl Zeiss). Blinded quantification was performed using ImageJ software (version 2.1.0/1.53c; National Institutes of Health). For immunofluorescences, the integrated density of pixels was measured for each section and a mean value was calculated as previously described.^22,30^ Maximum background threshold was determined for each image and set for intensity quantification. Data represent the mean ± standard error of the mean (SEM).

### RNA extraction, reverse transcription, and quantitative polymerase chain reaction

Total RNA was extracted from spinal cord segments (∼2 mm) either rostral or caudal to the lesion epicenter using TRIzol reagent (ThermoFisherScientific), following the manufacturer's instructions and methods previously described.^22,31^ Total RNA concentrations were determined by absorbance at 260 nm using a Nanodrop spectrophotometer (ThermoFisherScientific). RNA was reverse-transcribed into complementary DNA (cDNA) using Omniscript reverse transcriptase (Qiagen, Hilden, Germany). PowerUp SYBR Green Master Mix (ThermoFisherScientific) was used to measure mRNAs of interest by qPCR. Primers were designed using the Primer Blast program from NCBI (Table 3). Formation of single SYBR Green-labeled PCR amplicons was verified by running melting curve analysis. Threshold cycles (CTs) for each PCR reaction were identified by using QuantStudio 12K Flex software. To construct standard curves, serial dilutions were used from 1/2 to 1/512 of a pool of cDNAs generated by mixing equal amounts of cDNA from each sample. The CTs from each sample were compared to the relative standard curve to estimate the mRNA content per sample; the values obtained were then normalized using peptidylprolyl isomerase A (Ppia) mRNA.

**Table 3.** Primer Sequence for RT-qPCR

***Gene***	***Forward primer***	***Reverse primer***	***Product length (bp)***	***RefSeq***
Ppia	TGGTCAACCCCACCGTGTT	CCACCCTGGCACATGAATCCT	193	NM_008907.2
Slc17a7	CCCCATCATCGTGGGTGCAA	AGCTGGTCGTGGCCAACAAA	192	NM_182993
Grik1	TGTTCTGGCTGCAGGACTCG	GTGGAGTTGGTCGGATGGGT	133	NM_001346964.2

RT-qPCR, reverse-transcription quantitative polymerase chain reaction; Ppia, peptidylprolyl isomerase A; bp, base pair.

### High-pressure liquid chromatography lipid analysis

Spinal cord segments (∼2 mm) above and below the T9 region were collected from sham and SCI animals at 14 days post-SCI or laminectomy. Flash-frozen samples were used for lipid extraction, followed by anion-exchange high-pressure liquid chromatography (HPLC) quantification, as described previously.^23,32^

### Transcriptomic profiling by RNA sequencing

Total RNA was extracted from spinal cord segments above and below the injury (∼2 mm) from male mice at 14 days (*N* = 3 per group). RNA integrity was checked using the RNA 6000 Nano assay (Agilent Technologies, Santa Clara, CA). The sequencing library was prepared with a standard TruSeq RNA Sample Prep Kit v2 protocol (Illumina, San Diego, CA), as described previously.^22,33^ RNA libraries were sequenced on the Illumina HiSeq 2000 System with 100 nucleotide paired-end reads, according to the standard manufacturer's protocol (Illumina). For RNA-seq data analysis, Star 2.5.4b and bowtie2 2.1.0, samtools 0.1.7 and cufflinks 1.3.0 were used as described previously.^33–35^ Before read alignment, we identified the samples with the lowest read counts above and below the injury site (32630698 and 32116310, respectively) and downsampled all other samples of the same site to the same number of read counts by randomly removing excessive reads (Supplementary Fig. S3A) to prevent distortion of upper quartile normalization and RNA-seq results by read count imbalances, as described previously.^34^ The percentages of reads that were successfully aligned to the mouse reference genome varied between 85% and 89% (Supplementary Fig. S3B).

Differentially expressed genes (DEGs) between all four conditions at each site of the injury were identified based on a maximum *p* value of 0.01 (Supplementary Table S1). Up- and downregulated genes were submitted to pathway enrichment analysis using Fisher's exact test and the Gene Ontology Biological Processes 2018 library^36,37^ downloaded from enrichR^38^ (Supplementary Table S2). Predicted subcellular processes were ranked by significance. Significance *p* values were transformed into -log_10_(*p* values) and visualized as bar diagrams.

### Database mining

Information about the subcellular expression of Synj1 in the spinal cord of mice was obtained by data mining the harmonized atlas of spinal cord cells before and after SCI as previously described in the seqseek.ninds.nih.gov website.^39^

### Statistical analysis

Statistical evaluations were performed using *t*-tests for two comparisons or two-way mixed-model analysis of variance (ANOVA) and Tukey's multiple comparison test *post hoc*, where appropriate, as indicated in the figure legends. All statistical tests were two-tailed; *p* values of <0.05 were considered significant. Statistical calculations were performed using Prism software (version 9; GraphPad Software Inc., La Jolla, CA). Data are expressed as mean values ± SEM.

## Results

### Baseline phosphoinositol 4,5-bisphosphate levels are reduced in spinal cords of apolipoprotein E epsilon 4 mice

**FIG. 2.**
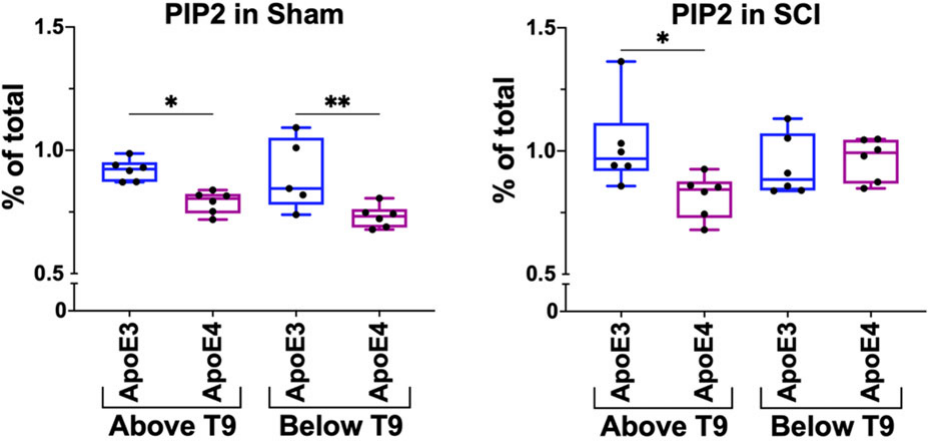
PIP_2_ levels in spinal cords of ApoE3 and ApoE4 mice after SCI. HPLC was used to determine levels of PIP_2_ in spinal cord tissue located above or below T9. Data are expressed as percentage of total lipid detected in the sample. Tissue was isolated at day 14 after a 50-kdyne contusion SCI (right panel) or sham SCI (left panel). *p < 0.05; **p < 0.01, two-way ANOVA. *N* = 6 per group. ANOVA, analysis of variance; ApoE3, apolipoprotein E epsilon 3; ApoE4, apolipoprotein E epsilon 4; HPLC, high-pressure liquid chromatography; PIP_2_, phosphoinositol 4,5-bisphosphate; SCI, spinal cord injury.

HPLC analysis of lipid profiles of spinal cord from sham ApoE4 animals showed lower baseline PIP_2_ levels both above and below T9 as compared to sham ApoE3 mice (Fig. 2). These studies were repeated using 2-mm segments of spinal cord collected 14 days after a 50-kdyne spinal cord contusion at T9 and were located just above or below the lesion epicenter. After SCI, PIP_2_ levels in ApoE4 mice were significantly lower above the lesion site when compared to ApoE3 mice. In contrast, PIP_2_ levels in spinal cord tissue below the level of the injury increased after SCI in ApoE4 mice to levels that were comparable to those found in ApoE3 mice (Fig. 2).

**FIG. 3.**
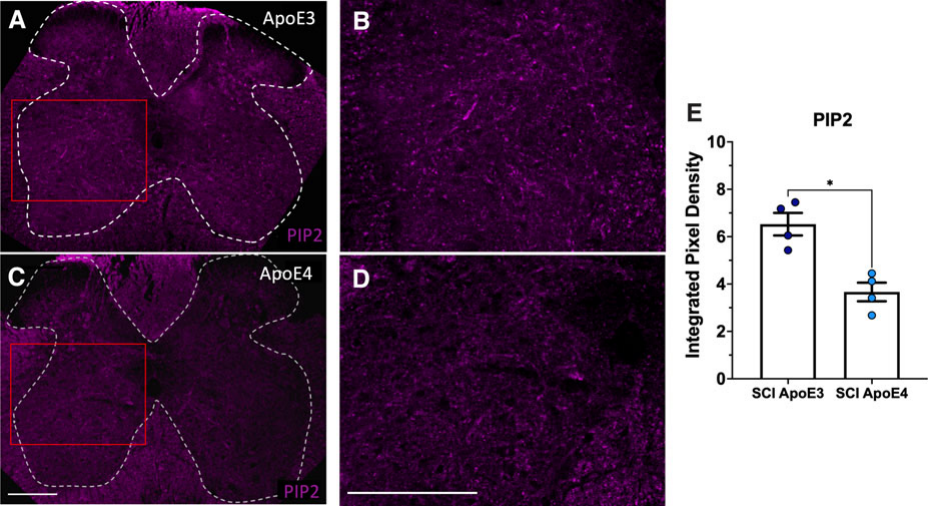
ApoE4 reduced PIP_2_ above the lesion at 14 dpi. Vibratome sections of perfusion-fixed spinal cords from ApoE3 (**A,B**) or ApoE4 (**C,D**) mice located at ∼300 microns above the injury epicenter were immunostained. Panels B and D show enlarged views of fields indicated in panels A and C by red boxes. (**E**) Fluorescence intensity was quantified for each of 4 different animals per ApoE variant. Images displayed are representative examples. *p < 0.01; unpaired Student's *t*-test. ApoE3, apolipoprotein E epsilon 3; ApoE4, apolipoprotein E epsilon 4; PIP_2_, phosphoinositol 4,5-bisphosphate; SCI, spinal cord injury.

To validate these biochemical studies, transverse 20-micron fixed sections of fixed spinal cord were collected and analyzed at 14 days post-injury. Sections from just above the lesion from ApoE3 or ApoE4 mice were subjected to immunofluorescence staining and visualized by confocal microscopy. Reduced levels of PIP_2_ were observed in sections from ApoE4 mice as compared to sections from ApoE3 mice from a location equidistant from the lesion (Fig. 3).

### Synaptojanin 1 levels are increased in spinal cord gray matter of apolipoprotein E epsilon 4 mice after SCI

**FIG. 4.**
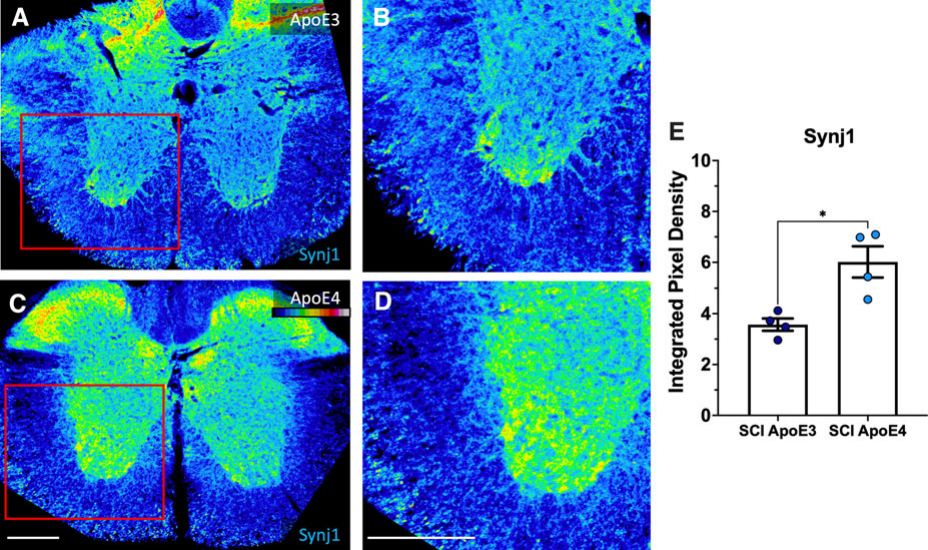
Synj1 levels were higher in tissues just above the lesion in ApoE4 mice at 14 dpi. (**A–D**). Immunostaining intensity for synj1 is depicted using a colorized grayscale for which the legend is shown in the top right corner of panel C. Fluorescence intensity was quantified (**E**). See Figure 3 for additional details. ApoE3, apolipoprotein E epsilon 3; ApoE4, apolipoprotein E epsilon 4; dpi, days post-injury; SCI, spinal cord injury; synj 1, synaptojanin 1.

Additional sections of spinal cords collected at 14 days after SCI were used to detect Synj1 by immunofluorescent staining. When examining sections taken just above the lesion, Synj was increased in ApoE4 mice as compared to ApoE3 mice (Fig. 4). Elevated Synj1 expression was observed mainly in the gray matter of sections from ApoE4 mice as compared to ApoE3 sections (Fig. 4).

### Genetically reducing synaptojanin 1 improves locomotor function in male apolipoprotein E epsilon 4 mice

**FIG. 5.**
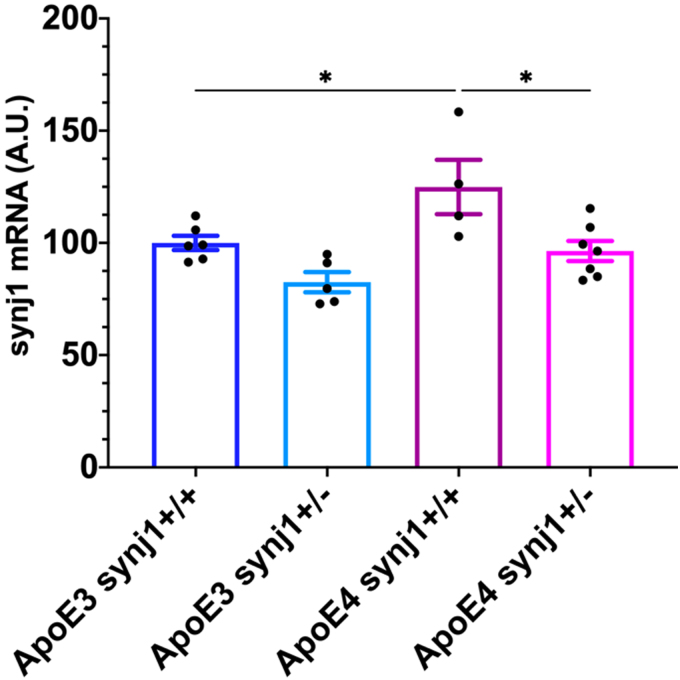
Changes in synj1 levels in spinal cords of male mice at 14 dpi. mRNA levels were determined by RT-qPCR after total RNA extraction from 4-mm spinal cord segments just above the lesion site. ApoE3 and ApoE4 mice either homozygous (synj1^+/+^) or heterozygous (synj1^+/–^) for synj1 were used. Data are shown as mean ± SEM. *p < 0.05, by two-way ANOVA. *N* = 4–7. ANOVA, analysis of variance; ApoE3, apolipoprotein E epsilon 3; ApoE4, apolipoprotein E epsilon 4; A.U., arbitrary units; dpi, days post-injury; mRNA, messenger RNA; qPCR, quantitative polymerase chain reaction; RT, reverse transcription; SCI, spinal cord injury; SEM, standard error of the mean; synj 1, synaptojanin 1.

**FIG. 6.**
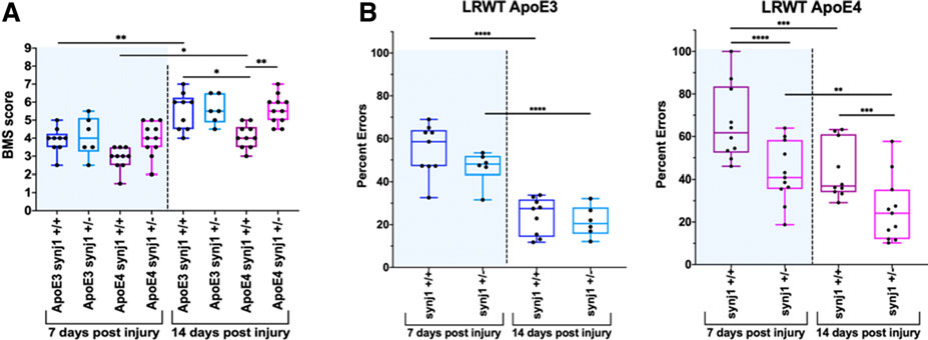
Genetic reduction of synj1 improves locomotion in male ApoE4 mice after SCI. (**A**) BMS scores were determined at 7 and 14 dpi for male mice with the indicated genotypes. (**B**) LWRT was performed at 7 and 14 dpi for male mice with the indicated genotypes. Results are expressed as percent of total hindlimb foot placement errors (steps that were missed, skipped, or slipped). **p < 0.01; ***p < 0.001; ****p < 0001, two-way ANOVA. *N* = 6–11. ANOVA, analysis of variance; ApoE3, apolipoprotein E epsilon 3; ApoE4, apolipoprotein E epsilon 4; BMS, Basso Mouse Scale; dpi, days post-injury; LWRT, ladder rung walk test; SCI, spinal cord injury; synj 1, synaptojanin 1.

We tested the effects of genetically reducing synj1 levels on outcomes after SCI in ApoE4 and ApoE3 mice. Animals expressing human ApoE4 that either had one inactive synj1 allele (synj1^+/–^) or two normal synj1 alleles (synj1^+/+^) were subjected to a motor-incomplete spinal cord contusion at the level of T9. RT-qPCR analysis showed that synj1 mRNA levels were 24.9% higher in ApoE4 synj1^+/+^ mice compared to ApoE3 synj1^+/+^ SCI mice (Fig. 5). This is consistent with the reported effect of ApoE4 to raise synj1 levels in the brain.^23^ In ApoE4 synj1^+/–^ mice, synj1 mRNA levels were reduced by 22.8% compared to ApoE4 synj1^+/+^ mice, confirming partial knockdown of this gene (Fig. 5). Open-field BMS testing was performed by two blinded scorers to evaluate locomotor function at 7 and 14 days post-injury (dpi; Fig. 6A). Horizontal LRWT was performed by blinded scorers using videos recorded with a GoPro camera, as previously reported,^22^ to evaluate hindlimb fine motor skills and coordination^28^ (Fig. 6B).

As expected, before surgery/laminectomy, all animals had maximum BMS scores, indicating normal hindlimb function. When compared to ApoE4 synj1^+/+^ at 14 dpi, ApoE4 synj1^+/–^ SCI mice had significantly higher BMS scores and significantly fewer hindfoot placement errors assessed by LRWT (Fig. 6). In contrast, BMS scores and LWRT in ApoE3 mice were not altered by the hemizygous inactivation of synj1 (Fig. 6). These findings support our hypothesis that reducing synj1 counteracts the deleterious effects of ApoE4 on function after SCI.

### Genetic reduction of synaptojanin 1 in apolipoprotein E epsilon 4 mice does not affect white spared matter after moderate-severity contusion spinal cord injury

To determine whether reduced levels of Synj1 affect white spared matter after injury in ApoE4 mice, we performed fluorescent myelin detection (Fluoromyelin; ThemoFisherScientific) at 14 days and used confocal imaging on spinal cord transverse sections from ApoE4 synj1^+/+^ and ApoE4 synj1^+/–^ at 150 μm above (rostral) and below (caudal) the injury site, as well as in the injury epicenter (Supplementary Fig. S3). The data suggest that Synj1 reduction does not affect white spared matter at 14 days after SCI.

### RNA sequencing reveals specific messenger RNA signatures in apolipoprotein E epsilon 4 synj1^+/–^ mice

**FIG. 7.**
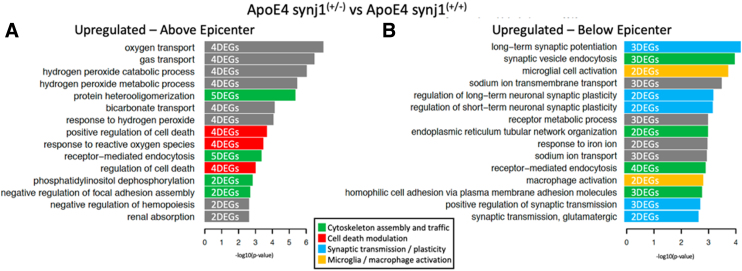
Differences in transcriptomic profiles for ApoE4 SCI male mice with either wild-type (synj1^+/+^) or genetically reduced (synj1^+/–^) synj1 at 14 dpi. DEGs (*p* ≤ 0.01) identified when comparing ApoE4 synj1^+/–^, and ApoE4 synj1^+/+^ was subjected to pathway enrichment analysis using Gene Ontology Biological Processes and Fisher's exact test, followed by a ranking of predicted pathways by significance. Top 15 ranked pathways that were either upregulated above epicenter (**A**) or upregulated below epicenter (**B**) for ApoE4 synj1(^+/-^). The indicated DEG counts document how many upregulated genes were part of each particular pathway. ApoE4, apolipoprotein E epsilon 4; DEGs, differentially expressed genes; dpi, days post-injury; SCI, spinal cord injury; synj 1, synaptojanin 1.

To obtain information regarding the potential molecular mechanisms responsible for improved functional outcomes after SCI in ApoE4 synj1^+/–^ mice, bulk RNA-seq was performed using total RNA isolated from spinal cord segments just above or below the lesion epicenter. Bioinformatics analysis of these RNA-seq data was performed as previously described.^22,35^ Analysis of differentially expressed mRNAs identified when comparing ApoE4 synj1^+/–^ and ApoE4 synj1^+/+^ mice at 14 dpi revealed that the most highly ranked Gene Ontology Biological Processes identified above the injury site differed from those below the injury site, indicating a spatially complex effect of the reduction of synj1 on cellular adaptations to the SCI (Fig. 7). Clear effects of reducing synj1 expression were observed on Biological Processes involved with synaptogenesis and axon extension below the lesion in ApoE4 synj1^+/–^ mice when compared to ApoE4 synj1^+/+^ controls (Fig. 7). Biological Processes represented by genes that were upregulated above the lesion included several involved in responses to or the metabolism of reactive oxygen species (Fig. 7).

**FIG. 8.**
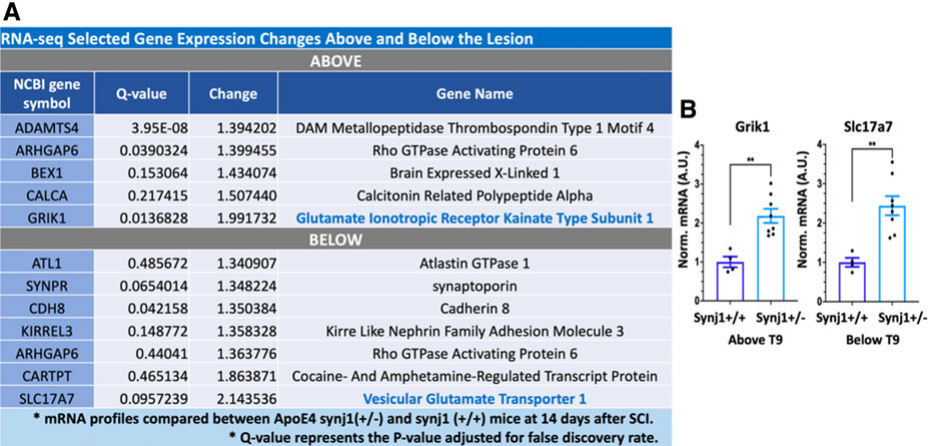
Selected gene expression changes between ApoE4 synj1 (+/+) and ApoE4 synj1 (+/–) male mice after SCI. (**A**) RNA-seq transcriptomic profile showed significant differences in expression of genes above and below the lesion site at 14 days. (**B**) RT-qPCR was performed to validate changes in Grik1 and Slc17a7 levels after SCI in male mice using total RNA isolated from spinal cord segments above and below the injury. Tissues from ApoE4 mice that were either homozygous (synj1^+/+^) or heterozygous (synj1^+/–^) for synj1 were collected at 14 days to validate RNA-seq studies. Bar plots display mean ± SEM. **p < 0.01, unpaired Student's *t*-test. *N* = 4–8. ApoE4, apolipoprotein E epsilon 4; qPCR, quantitative polymerase chain reaction; RNA-seq, RNA sequencing; RT, reverse transcription; SCI, spinal cord injury; SEM, standard error of the mean; synj 1, synaptojanin 1.

We examined the lists of the most highly altered DEGs to better understand how lowering synj1 improved function in ApoE4 mice after SCI (Fig. 7). One of these was GRIK1, a subunit of glutaminergic ionotropic kainite receptors that was altered above the lesion (Supplementary Table S1; Fig. 7). The second highly differentially expressed gene was SLC17A7, which transports glutamate into synaptic vesicles and was altered below the lesion (Fig. 7). Other genes involved in stabilizing synapses were upregulated below the lesion, including KIRREL3 and cadherin 8 (Supplementary Table S1). ARHGAP6, a gene involved in extending cellular projections, was upregulated above and below the lesion (Supplementary Table S1); ATL1, a gene involved in axon maintenance, was increased below the lesion (Supplementary Table S1).

### Cell-type–specific expression profiles of synaptojanin 1 in mice spinal cord

To better understand the cell-type–specific expression of synj1 in spinal cord, we mined the harmonized atlas of spinal cord cells post-SCI.^39,40^ We first searched for expression levels of synj1 in all cells represented in the atlas, which reports mRNA expression levels in cells within the lumbar spinal cord at times from 1 day to 6 weeks after a spinal cord contusion. Synj1 was expressed predominantly in neurons, with astrocytes, microglia, and oligodendrocytes also showing expression. Smaller numbers of other cell types were observed to express some synj1 (Supplementary Fig. S4). Synj1 expression levels did not appear to be altered by SCI (Supplementary Fig. S5).

## Discussion

The major conclusion supported by this study is that, at 14 days post-injury, genetically reducing expression levels of synj1 increases locomotor functional recovery after a 50-kdyne contusion SCI at T9 in male ApoE4 TR mice, but not in ApoE3 TR mice. The data also support the conclusion that in uninjured TR mice, ApoE4 increased synj1 mRNA expression and reduced levels of PIP_2_ in spinal cord homogenates when compared to mice with a TR for ApoE3.

In the literature, synj1 is reported to be most highly expressed in neurons and to be concentrated at synapses consistent with its role in trafficking of synaptic membranes.^24^ In agreement with these studies of the brain, we found by immunostaining of spinal cord sections that the most intense staining for synj1 was in the gray matter. Our database mining suggests that in spinal cord, synj1 is expressed most strongly in neurons, with some expression in astrocytes, microglia, and olidodendrocytes, and that expression levels are relatively constant over time after SCI. Each of these cell types plays critical roles in recovery of function after SCI.^41–44^ The database contained snSEQ data obtained at 1 day to 6 weeks after a midthoracic contusion SCI for samples of spinal cord from the lumbar region, a site caudal to the lesion. Thus, some caution should be used when extrapolating the findings to other spinal cord segments, but the general principle that synj1 is highly expressed in neurons of gray matter of the spinal cord and found in low abundance in glia is likely to hold true for all segments of spinal cord.

The mechanisms by which synj1 upregulation in ApoE4 mice blunts locomotor functional recovery are unclear. The finding that genetic reduction of synj1 did not alter white matter sparing in ApoE4 mice indicates that tissue damage post-SCI was not affected by this genetic manipulation. The neuroanatomical basis of the effect of synj1 on the locomotor function of ApoE4 mice must therefore depend on surviving circuits that involve either formation of relay circuits through axon branching or greater synaptic function of the remaining motor circuitry. The available data do not allow us to distinguish between these possibilities, which are not mutually exclusive. Links between synj1 levels and synaptic function have been reported.^24^ Although no links between synj1 and neurite outgrowth have been reported, synj1 is well known to determine vesicle trafficking and cytoskeletal rearrangements, and our experimentally validated computational modeling has established links between vesicular transport and neurite outgrowth.^45^ Synj1 is also involved in synaptic function, and it remains possible that excessive synj1 expression impairs synaptic function in neural circuits that support locomotor function after SCI. This interpretation of the data is consistent with our findings that reducing synj1 expression did not increase spared white matter, though further experimentation is needed to delineate the respective roles of increased formation of relay circuits from improved synaptic transmission within spared neural circuits.

An unexpected finding was the marked difference in the DEGs observed when comparing bulk RNA-seq data for tissues above versus below the injury epicenter. Most of the DEGs identified below the lesion were involved in synaptic transmission or other functions of neurons whereas none of the DEGs identified above the lesion functioned in synaptic transmission. It should be noted that only a small number of DEGs were identified for each gene ontology, and that it is possible that there are DEGs for synaptic transmission or axonal growth that are altered by reducing synj1 in ApoE4 mice at both sites that were not detected because of the sample size used in these experiments.

A second unexpected finding was that genetically reducing the expression of synj1 in TR ApoE3 mice did not seem to alter functional recovery after SCI. The implication of this result is that findings of studies of genetic manipulation in ApoE4 mice are not necessarily generalizable to mice with other ApoE backgrounds and that further study is needed to better understand the barriers to maximal functional recovery for each ApoE genotype. Of note, it becomes necessary to conduct additional studies at longer time points in order to confirm the final status of functional recovery for all genotypes involved. In addition, our study did not include females. Even though males account for 80% of persons with SCI and near 97% of veterans with SCI are males,^1,46^ it is necessary to conduct additional studies using female ApoE4 mice expressing normal or reduced levels of synj1 to test for any possible sex differences, given that sex has been reported to be an important biological variable in AD pathogenesis.^47^ Of note, our previous work did not show any sex differences between ApoE4 mice after incomplete SCI,^22^ leading us to speculate that any sex discordance that may be observed is unlikely to be large.

Our findings suggest that there is a high likelihood that effective, personalized therapies could be developed to improve functional recovery of those who have a new SCI that involve manipulating levels of genes dysregulated by commonly occurring genetic variants, or therapies that regulate activities of the proteins they encode. This possibility provides a strong rationale for continued study of the differential effects of ApoE variants on functional recovery after SCI and on the underlying molecular basis for the differences observed. Such studies may ultimately result in new treatments to improve the function of persons recovering from SCI.

## Supplementary Material

Supplemental data

Supplemental data

Supplemental data

Supplemental data

Supplemental data

Supplemental data

Supplemental data

## Data Availability

The data are available upon written, reasonable request. Gene Expression Omnibus (GEO) data accession GSE227291.

## Abbreviations Used

AD: Alzheimer's disease
ANOVA: analysis of variance
ApoE: apolipoprotein E
ApoE4: apolipoprotein E epsilon 4
BMS: Basso Mouse Scale
cDNA: complementary DNA
CTs: threshold cycles
DEGs: differentially expressed genes
dpi: days post-injury
HPLC: high-pressure liquid chromatography
IH: Infinite Horizons
LRWT: ladder rung walk test
mRNA: messenger RNA
PCR: polymerase chain reaction
PFA: paraformaldehyde
PIP_1_: inositol 4-monophosphate
PIP_2_: phosphoinositol 4,5-bisphosphate
Ppia: peptidylprolyl isomerase A
qPCR: quantitative PCR
RNA-seq: RNA sequencing
SCI: spinal cord injury
SEM: standard error of the mean
synj 1: synaptojanin 1

## References

[1] National Spinal Cord Injury Statistical Center. Spinal Cord Injury (SCI) 2020 Facts and Figures at a Glance. National Spinal Cord Injury Statistical Center: Birmingham, AL; 2020.

[2] Basso DM, Beattie MS, Bresnahan JC. Graded histological and locomotor outcomes after spinal cord contusion using the NYU weight-drop device versus transection. Exp Neurol 1996;139(2):244–256; doi: 10.1006/exnr.1996.0098 8654527

[3] Courtine G, Song B, Roy RR, et al. Recovery of supraspinal control of stepping via indirect propriospinal relay connections after spinal cord injury. Nat Med 2008;14(1):69–74; doi: 10.1038/nm1682 18157143 PMC2916740

[4] Asboth L, Friedli L, Beauparlant J, et al. Cortico-reticulo-spinal circuit reorganization enables functional recovery after severe spinal cord contusion. Nat Neurosci 2018;21(4):576–588; doi: 10.1038/s41593-018-0093-5 29556028

[5] Bareyre FM, Kerschensteiner M, Raineteau O, et al. The injured spinal cord spontaneously forms a new intraspinal circuit in adult rats. Nat Neurosci 2004;7(3):269–277; doi: 10.1038/nn1195 14966523

[6] Hakim R, Zachariadis V, Sankavaram SR, et al. Spinal cord injury induces permanent reprogramming of microglia into a disease-associated state which contributes to functional recovery. J Neurosci 2021;41(40):8441–8459; doi: 10.1523/jneurosci.0860-21.2021 34417326 PMC8496189

[7] Renault-Mihara F, Okada S, Shibata S, et al. Spinal cord injury: emerging beneficial role of reactive astrocytes' migration. Int J Biochem Cell Biol 2008;40(9):1649–1653; doi: 10.1016/j.biocel.2008.03.009 18434236

[8] Schwab JM, Zhang Y, Kopp MA, et al. The paradox of chronic neuroinflammation, systemic immune suppression, autoimmunity after traumatic chronic spinal cord injury. Exp Neurol 2014;258:121–129; doi: 10.1016/j.expneurol.2014.04.023 25017893 PMC4099970

[9] Fu H, Zhao Y, Hu D, et al. Depletion of microglia exacerbates injury and impairs function recovery after spinal cord injury in mice. Cell Death Dis 2020;11(7):528; doi: 10.1038/s41419-020-2733-4 32661227 PMC7359318

[10] Filli L, Engmann AK, Zorner B, et al. Bridging the gap: a reticulo-propriospinal detour bypassing an incomplete spinal cord injury. J Neurosci 2014;34(40):13399–13410; doi: 10.1523/JNEUROSCI.0701-14.2014 25274818 PMC6608315

[11] Liu CC, Liu CC, Kanekiyo T, et al. Apolipoprotein E and Alzheimer disease: risk, mechanisms and therapy. Nat Rev Neurol 2013;9(2):106–118; doi: 10.1038/nrneurol.2012.263 23296339 PMC3726719

[12] Rebeck GW, Reiter JS, Strickland DK, et al. Apolipoprotein E in sporadic Alzheimer's disease: allelic variation and receptor interactions. Neuron 1993;11(4):575–580. 8398148 10.1016/0896-6273(93)90070-8

[13] Ariza M, Pueyo R, Matarín Mdel M, et al. Influence of APOE polymorphism on cognitive and behavioural outcome in moderate and severe traumatic brain injury. J Neurol Neurosurg Psychiatry 2006;77(10):1191–1193; doi: 10.1136/jnnp.2005.085167 16614010 PMC2077553

[14] Crawford FC, Vanderploeg RD, Freeman MJ, et al. APOE genotype influences acquisition and recall following traumatic brain injury. Neurology 2002;58(7):1115–1118. 11940706 10.1212/wnl.58.7.1115

[15] Liberopoulos E, Siamopoulos K, Elisaf M. Apolipoprotein E and renal disease. Am J Kidney Dis 2004;43(2):223–233; doi: 10.1053/j.ajkd.2003.10.013 14750087

[16] Mahley RW. Apolipoprotein E: from cardiovascular disease to neurodegenerative disorders. J Mol Med (Berl) 2016;94(7):739–746; doi: 10.1007/s00109-016-1427-y 27277824 PMC4921111

[17] Conejero-Goldberg C, Gomar JJ, Bobes-Bascaran T, et al. APOE2 enhances neuroprotection against Alzheimer's disease through multiple molecular mechanisms. Mol Psychiatry 2014;19(11):1243–1250; doi: 10.1038/mp.2013.194 24492349

[18] Shinohara M, Kanekiyo T, Yang L, et al. APOE2 eases cognitive decline during aging: clinical and preclinical evaluations. Ann Neurol 2016;79(5):758–774; doi: 10.1002/ana.24628 26933942 PMC5010530

[19] Li Z, Shue F, Zhao N, et al. APOE2: protective mechanism and therapeutic implications for Alzheimer's disease. Mol Neurodegener 2020;15(1):63; doi: 10.1186/s13024-020-00413-4 33148290 PMC7640652

[20] Jha A, Lammertse DP, Coll JR, et al. Apolipoprotein E epsilon4 allele and outcomes of traumatic spinal cord injury. J Spinal Cord Med 2008;31(2):171–176. 18581664 10.1080/10790268.2008.11760708 PMC2565476

[21] Desimone A, Hong J, Brockie ST, et al. The influence of ApoE4 on the clinical outcomes and pathophysiology of degenerative cervical myelopathy. JCI Insight 2021;6(15):e149227; doi: 10.1172/jci.insight.149227 34369386 PMC8410082

[22] Toro CA, Hansen J, Siddiq MM, et al. The human ApoE4 variant reduces functional recovery and neuronal sprouting after incomplete spinal cord injury in male mice. Front Cell Neurosci 2021;15:626192; doi: 10.3389/fncel.2021.626192 33679326 PMC7930340

[23] Zhu L, Zhong M, Elder GA, et al. Phospholipid dysregulation contributes to ApoE4-associated cognitive deficits in Alzheimer's disease pathogenesis. Proc Natl Acad Sci U S A 2015;112(38):11965–11970; doi: 10.1073/pnas.1510011112 26372964 PMC4586834

[24] Choudhry H, Aggarwal M, Pan P-Y. Mini-review: synaptojanin 1 and its implications in membrane trafficking. Neurosci Lett 2021;765:136288; doi: 10.1016/j.neulet.2021.136288 34637856 PMC8572151

[25] Mandal K. Review of PIP_2_ in cellular signaling, functions and diseases. Int J Mol Sci 2020;21(21):8342; doi: 10.3390/ijms21218342 33172190 PMC7664428

[26] Scheff SW, Rabchevsky AG, Fugaccia I, et al. Experimental modeling of spinal cord injury: characterization of a force-defined injury device. J Neurotrauma 2003;20(2):179–193; doi: 10.1089/08977150360547099 12675971

[27] Basso DM, Fisher LC, Anderson AJ, et al. Basso Mouse Scale for locomotion detects differences in recovery after spinal cord injury in five common mouse strains. J Neurotrauma 2006;23(5):635–659; doi: 10.1089/neu.2006.23.635 16689667

[28] Cummings BJ, Engesser-Cesar C, Cadena G, et al. Adaptation of a ladder beam walking task to assess locomotor recovery in mice following spinal cord injury. Behav Brain Res 2007;177(2):232–241; doi: 10.1016/j.bbr.2006.11.042 17197044 PMC1892162

[29] Streijger F, Lee JH, Manouchehri N, et al. Responses of the acutely injured spinal cord to vibration that simulates transport in helicopters or mine-resistant ambush-protected vehicles. J Neurotrauma 2016;33(24):2217–2226; doi: 10.1089/neu.2016.4456 27214588

[30] Oliveira AL, Thams S, Lidman O, et al. A role for MHC class I molecules in synaptic plasticity and regeneration of neurons after axotomy. Proc Natl Acad Sci U S A 2004;101(51):17843–17848; doi: 10.1073/pnas.0408154101 15591351 PMC539738

[31] Toro CA, Wright H, Aylwin CF, et al. Trithorax dependent changes in chromatin landscape at enhancer and promoter regions drive female puberty. Nat Commun 2018;9(1):57; doi: 10.1038/s41467-017-02512-1 29302059 PMC5754362

[32] Cao J, Gaamouch FE, Meabon JS, et al. ApoE4-associated phospholipid dysregulation contributes to development of Tau hyper-phosphorylation after traumatic brain injury. Sci Rep 2017;7(1):11372; doi: 10.1038/s41598-017-11654-7 28900205 PMC5595858

[33] Mariottini C, Munari L, Gunzel E, et al. Wilm's tumor 1 promotes memory flexibility. Nat Commun 2019;10(1):3756; doi: 10.1038/s41467-019-11781-x 31434897 PMC6704057

[34] Stillitano F, Hansen J, Kong CW, et al. Modeling susceptibility to drug-induced long QT with a panel of subject-specific induced pluripotent stem cells. Elife 2017;6:e19406; doi: 10.7554/eLife.19406 28134617 PMC5279943

[35] Hansen J, Galatioto J, Caescu CI, et al. Systems pharmacology-based integration of human and mouse data for drug repurposing to treat thoracic aneurysms. JCI Insight 2019;4(11):e127652; doi: 10.1172/jci.insight.127652 31167969 PMC6629138

[36] Ashburner M, Ball CA, Blake JA, et al. Gene ontology: tool for the unification of biology. The Gene Ontology Consortium. Nat Genet 2000;25(1):25–29; doi: 10.1038/75556 10802651 PMC3037419

[37] Gene Ontology Consortium. The Gene Ontology resource: enriching a GOld mine. Nucleic Acids Res 2021;49(D1):D325–D334; doi: 10.1093/nar/gkaa1113 33290552 PMC7779012

[38] Chen EY, Tan CM, Kou Y, et al. Enrichr: interactive and collaborative HTML5 gene list enrichment analysis tool. BMC Bioinformatics 2013;14:128; doi: 10.1186/1471-2105-14-128 23586463 PMC3637064

[39] Matson KJE, Russ DE, Kathe C, et al. Single cell atlas of spinal cord injury in mice reveals a pro-regenerative signature in spinocerebellar neurons. Nat Commun 2022;13(1):5628; doi: 10.1038/s41467-022-33184-1 36163250 PMC9513082

[40] Russ DE, Cross RBP, Li L, et al. A harmonized atlas of mouse spinal cord cell types and their spatial organization. Nat Commun 2021;12(1):5722; doi: 10.1038/s41467-021-25125-1 34588430 PMC8481483

[41] Brennan FH, Li Y, Wang C, et al. Microglia coordinate cellular interactions during spinal cord repair in mice. Nat Commun 2022;13(1):4096; doi: 10.1038/s41467-022-31797-0 35835751 PMC9283484

[42] Okada S, Hara M, Kobayakawa K, et al. Astrocyte reactivity and astrogliosis after spinal cord injury. Neurosci Res 2018;126:39–43; doi: 10.1016/j.neures.2017.10.004 29054466

[43] Bellver-Landete V, Bretheau F, Mailhot B, et al. Microglia are an essential component of the neuroprotective scar that forms after spinal cord injury. Nat Commun 2019;10(1):518; doi: 10.1038/s41467-019-08446-0 30705270 PMC6355913

[44] Duncan GJ, Manesh SB, Hilton BJ, et al. The fate and function of oligodendrocyte progenitor cells after traumatic spinal cord injury. Glia 2020;68(2):227–245; doi: 10.1002/glia.23706 31433109

[45] Yadaw AS, Siddiq MM, Rabinovich V, et al. Dynamic balance between vesicle transport and microtubule growth enables neurite outgrowth. PLoS Comput Biol 2019;15(5):e1006877; doi: 10.1371/journal.pcbi.1006877 31042702 PMC6546251

[46] Curtin CM, Suarez PA, Di Ponio LA, et al. Who are the women and men in Veterans Health Administration's current spinal cord injury population? J Rehabil Res Dev 2012;49(3):351–360. 22773195 10.1682/jrrd.2010.11.0220

[47] Hsu M, Dedhia M, Crusio WE, et al. Sex differences in gene expression patterns associated with the APOE4 allele. F1000Res 2019;8:387; doi: 10.12688/f1000research.18671.2 31448102 PMC6685458

