# Supplementary material for: Synaptojanin 1 Modulates Functional Recovery After Incomplete Spinal Cord Injury in Male Apolipoprotein E Epsilon 4 Mice
Source: Neurotrauma Rep. 2023 Jul 27;4(1):464–77. doi: 10.1089/neur.2023.0023 (PMC10389254; doi:10.1089/neur.2023.0023)

**Supplementary Figure 1.** Body weights were determined on the day of surgery prior to making an incision (pre-op) and at the specified times shown on the X-axis for male mice. Data are shown as mean values ± SEM.


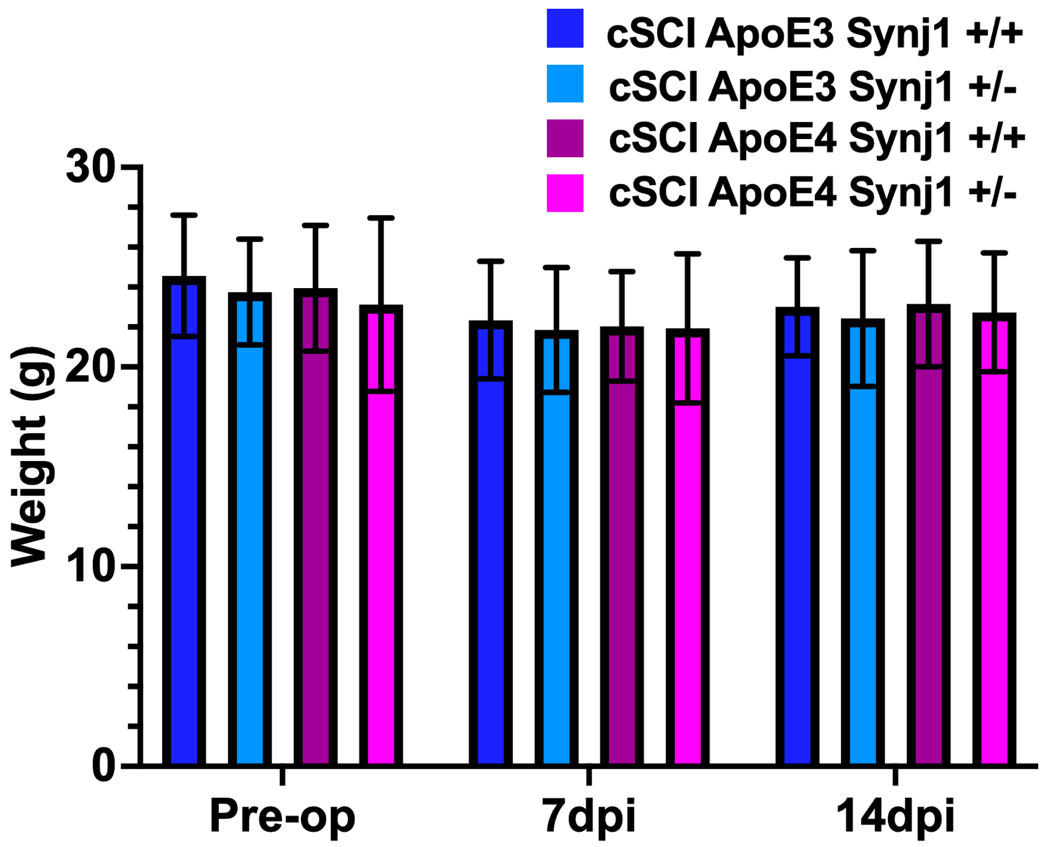

Supplement: Supplemental data [file Suppl_FigureS1.docx]
