# Supplementary material for: Synaptojanin 1 Modulates Functional Recovery After Incomplete Spinal Cord Injury in Male Apolipoprotein E Epsilon 4 Mice
Source: Neurotrauma Rep. 2023 Jul 27;4(1):464–77. doi: 10.1089/neur.2023.0023 (PMC10389254; doi:10.1089/neur.2023.0023)

**Supplementary Figure 2.** Actual impact force (kdyne) and spinal cord displacement during impact (µm) are shown. Data are shown as mean ± SEM.


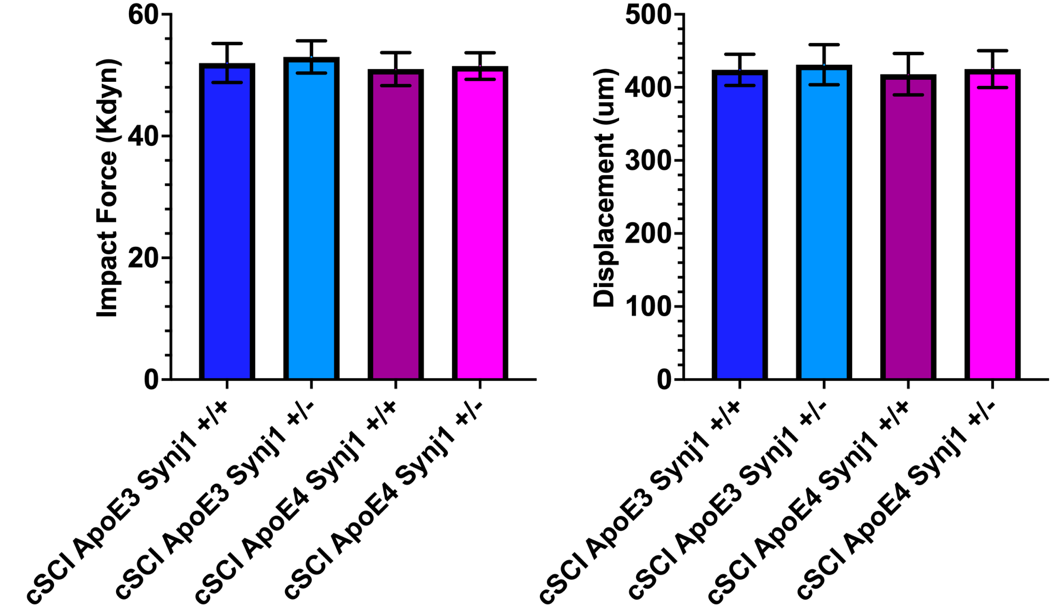

Supplement: Supplemental data [file Suppl_FigureS2.docx]
