# Supplementary material for: Synaptojanin 1 Modulates Functional Recovery After Incomplete Spinal Cord Injury in Male Apolipoprotein E Epsilon 4 Mice
Source: Neurotrauma Rep. 2023 Jul 27;4(1):464–77. doi: 10.1089/neur.2023.0023 (PMC10389254; doi:10.1089/neur.2023.0023)

**Supplementary Figure 3.** White spared matter is not affected by genetic reduction of Synj1 in ApoE4 male mice after SCI. A) Perfusion-fixed spinal cords cryosections from ApoE4 mice with either Syn1^+/+^ or Synj1^+/-^ were obtained at 14 days after SCI. Transverse sections were obtained rostral and caudal from the injury site as well as at epicenter. Sections were later stained with FluoroMyelin. Panel shows representative images at the epicenter and at 150 μm rostral and caudal from epicenter. Dashed lines outline sections and white spared matter (B) Quantification of myelin staining was performed for each section and showed no significant differences between ApoE4 synj1^+/+^ and ApoE4 synj1^+/-^. Bar plots are presented as mean ± SEM. N=2.


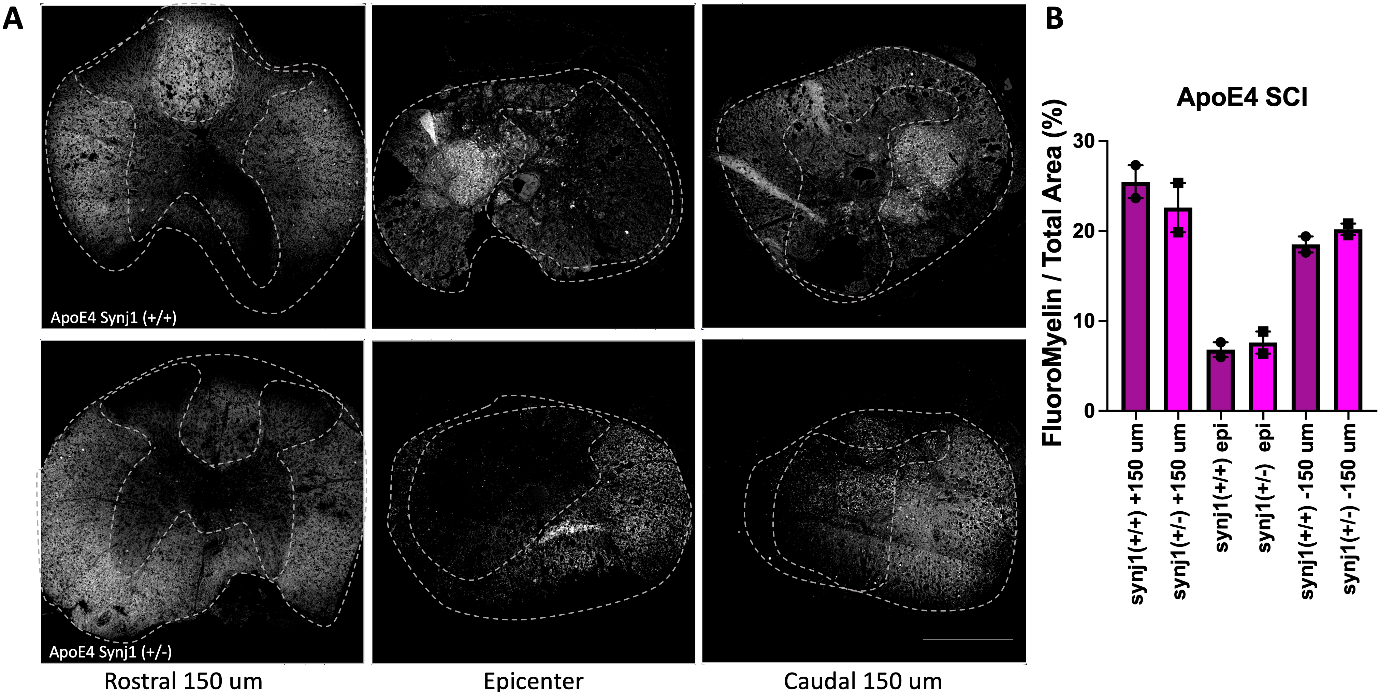

Supplement: Supplemental data [file Suppl_FigureS3.docx]
