# Supplementary material for: Synaptojanin 1 Modulates Functional Recovery After Incomplete Spinal Cord Injury in Male Apolipoprotein E Epsilon 4 Mice
Source: Neurotrauma Rep. 2023 Jul 27;4(1):464–77. doi: 10.1089/neur.2023.0023 (PMC10389254; doi:10.1089/neur.2023.0023)

**Supplementary Figure 4.** Synj1 is expressed different cellular subtypes within mouse spinal cords after SCI. Unsupervised snSEQ clustering after dimensionality reduction and cell neighborhood identification allowed the detection of Synj1 in all major cell types of the spinal cord. Graphs were obtained from data mining the website seqseek.ninds.nih.gov.


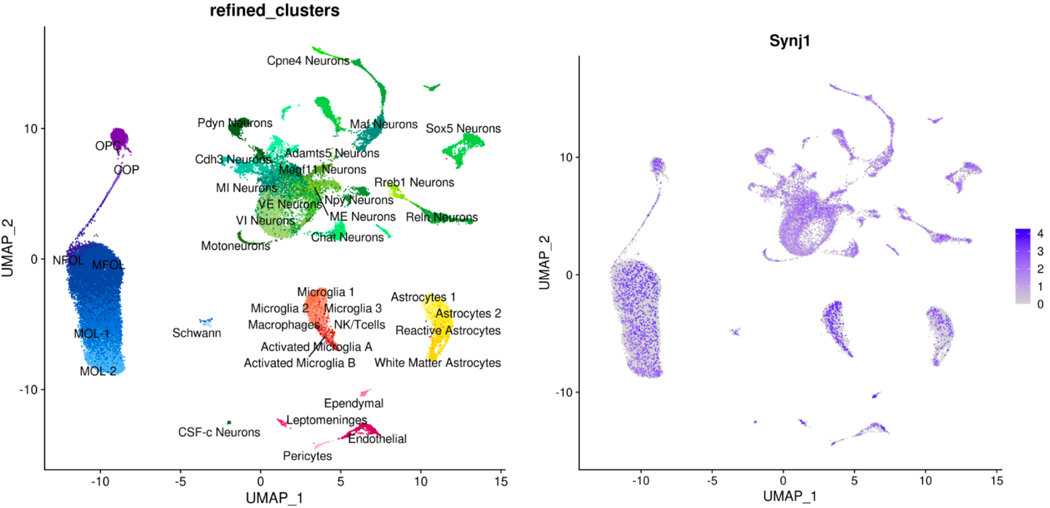

Supplement: Supplemental data [file Suppl_FigureS4.docx]
