# Supplementary material for: Synaptojanin 1 Modulates Functional Recovery After Incomplete Spinal Cord Injury in Male Apolipoprotein E Epsilon 4 Mice
Source: Neurotrauma Rep. 2023 Jul 27;4(1):464–77. doi: 10.1089/neur.2023.0023 (PMC10389254; doi:10.1089/neur.2023.0023)

**Supplementary Figure 5.** snSEQ profile of synj1 in mice spinal cord before and after SCI. Expression levels of Synj1 in different cell types during pre-surgery and after SCI at day 1, and at weeks 1, 3 and 6. Data mining was obtained from seqseek.ninds.nih.gov. website.


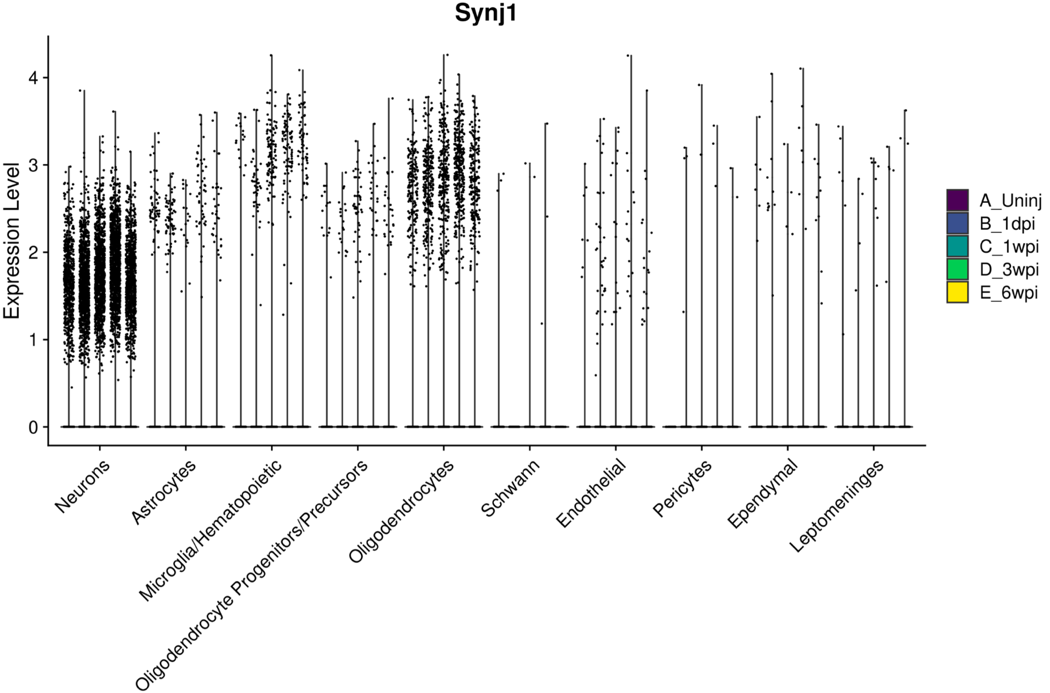

Supplement: Supplemental data [file Suppl_FigureS5.docx]
